# Complexes of Glycolic Acid with Nitrogen Isolated in Argon Matrices. II. Vibrational Overtone Excitations

**DOI:** 10.3390/molecules24183245

**Published:** 2019-09-06

**Authors:** Iwona Kosendiak, Jussi M.E. Ahokas, Justyna Krupa, Jan Lundell, Maria Wierzejewska

**Affiliations:** 1 Faculty of Chemistry, University of Wroclaw, Joliot-Curie 14, 50-383 Wroclaw, Poland; 2 Department of Chemistry, University of Jyvaskyla, P.O.Box 35, 40014 Jyvaskyla, Finland

**Keywords:** hydrogen bond, matrix isolation, carboxylic acid, computational chemistry, vibrational spectroscopy, vibrational overtone

## Abstract

Structural changes of glycolic acid (GA) complex with nitrogen induced by selective overtone excitation of the νOH mode were followed in argon matrices using FTIR spectroscopy. For the most stable SSC1 complex present in different trapping sites directly upon deposition site, selective changes in the νOH region were achieved upon near-infrared irradiation. Simultaneously, new conformers of the GA…N_2_ complex were formed, giving rise to several sets of bands in the νOH and νC=O regions of the spectra. Both position and intensity of new absorptions appeared to be highly sensitive on the wavelength of radiation used, as well as on the annealing of the matrix. Based on theoretical calculations at different levels of theory, an assignment of the observed bands is proposed and discussed.

## 1. Introduction

Weak interactions have an important contribution to chemical and photochemical processes taking place in the Earth’s atmosphere. Traditionally, photochemical processes are those requiring excitation of electronic states using UV radiation. Another mechanism for inducing photochemical reactions may be generated by near-infrared (NIR) or visible light promoting excitation of higher vibrational states [1,2,3]. The concept of near-infrared photochemistry was suggested by Donaldson et al. [1], for example, to describe OH-containing species such as HONO, HNO_3_, carboxylic acid, or alcohols absorbing NIR or visible radiation via vibrational overtones of the OH stretching mode that induced rotamerisation or even dissociation of the molecule. Up-to-now, the majority of overtone excitation studies has been reported for carboxylic acids [4,5,6,7,8,9,10,11,12,13]. As follows, glycolic acid (GA), the smallest α-hydroxycarboxylic acid, is also an advantageous object for such studies due to the presence of two hydroxyl groups with different surroundings in the same molecule.

**Figure 1:**
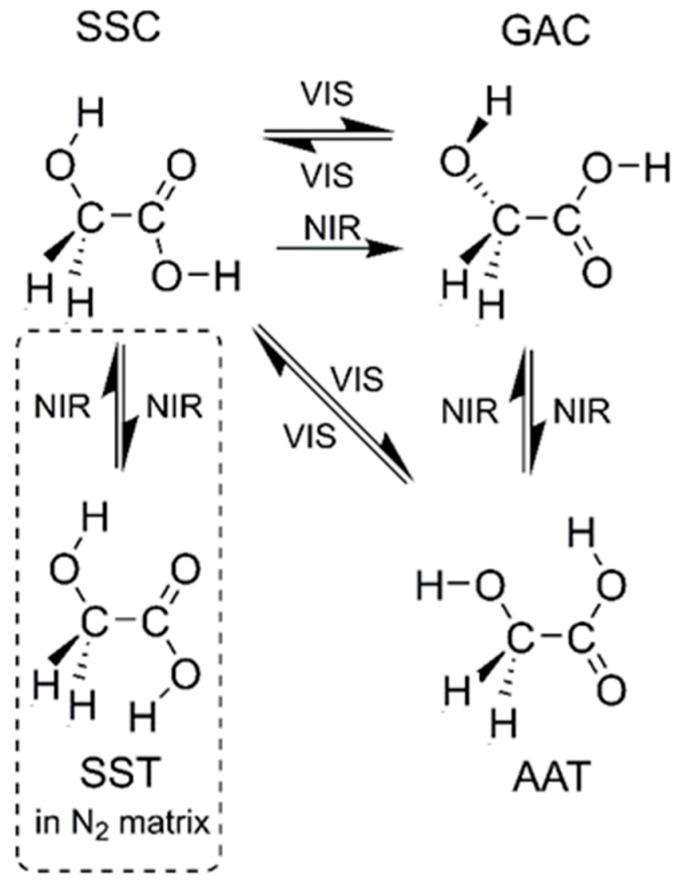
Light-induced conformational isomerization channels observed in low temperature matrices. The conformer abbreviations are those introduced in reference [6].

A number of studies have been performed on the first OH overtone excitation employing NIR radiation of the most stable conformer (SSC) of monomeric glycolic acid in low temperature matrices [4,6,14,15]. The observed photo-induced processes of the glycolic acid monomer are shown in Figure 1. Upon excitation of the first overtone of the OH stretching vibration, the SSC form converted in noble gas matrices into higher-energy GAC and AAT conformers: GAC was a preliminary product, whereas AAT was formed in a two-step process of SSC→GAC→AAT. Also, back reactions were observed. In turn, the high overtone excitation at 532 nm probed in conjunction with Raman spectroscopy [16] induced direct conformational isomerization to both higher energy conformers, GAC and AAT. In the same study, GAC and AAT were found to isomerize back to SSC, resulting finally to photo-induced equilibrium between the conformers.

Although the overtone-induced isomerisation of the monomeric GA is relatively well known, reports on isomerisation processes upon NIR radiation of its molecular complexes and the influence of complexation on these processes are scarce. Recently, we reported on conformational isomerisation of GA complex with nitrogen induced by high vibrational overtone excitation using green light (532 nm) in a study applying Raman spectroscopy [16]. On the contrary to the GA monomer, where both AAT and GAC conformers could be produced in argon matrices [4,6,12], only AAT…N_2_ complexes were identified at the applied experimental conditions [16].

Previously, we have studied the complexes between glycolic acid and molecular nitrogen [17], where GA was co-deposited with N_2_/Ar mixture. This experimental condition appeared to give specific molecular complexes which also represent the first step from pure Ar matrix towards full N_2_ environment. In GA/N_2_/Ar mixture, SSC…N_2_ complex was observed with the nitrogen molecule interacting with the carboxylic OH group (complex named SSC1) [17]. Here, we extend this preluding study by site-selective photo-induced chemistry via the first OH vibrational overtone excitations of GA…N_2_ complexes.

## 2. Methods 

### 2.1. Experimental Details

The matrix samples were prepared by passing mixtures of high purity argon (Messer, 5.0) and nitrogen (Messer, 6.0) with the N_2_/Ar ratios of 1/4000 through the glass U-tube with glycolic acid (GA) situated outside the cryostat chamber. The GA/N_2_/Ar gaseous mixtures were deposited onto a cold CsI window kept at 18 K in a closed cycle cryostat APD-Cryogenics (ARS-2HW). Conditions of the matrices’ deposition were fixed to obtain samples containing mostly monomeric GA and its complexes of the 1:1 stoichiometry. Low temperature was maintained by a Scientific Instruments 9700 temperature controller equipped with a silicon diode. FTIR spectra were collected at 10 K in a transmission mode with a 0.5 cm^−1^ resolution using a Bruker IFS 66 Fourier Transform spectrometer equipped with a liquid N_2_ cooled MCT detector.

Conformational changes in the GA/N_2_/Ar matrices were selectively induced by the NIR radiation provided by an Optical Parametric Oscillator (OPO) (Vibrant, Opotek Inc., Carlsbad, CA, USA) pumped with a Nd:YAG laser (Quantel) (7 ns, repetition rate of 10 Hz). The width of the Idler (NIR) output was *ca.* 7 cm^−1^, and the pulse energy was between 2.9–3.2 mJ as monitored by power and energy meter (Thorlabs) equipped with pyroelectric sensor.

### 2.2. Computational Details

All calculations were carried out using the Gaussian 16 program package [18]. In addition to the calculations reported earlier [17] at MP2 [19,20,21,22] and B3LYPD3 [23,24,25,26,27] levels employing the 6-311++G(2d,2p) basis set, the 1:1 complexes of the three most stable isomers (SSC, GAC, AAT) of GA with nitrogen were optimized using the B3LYPD3 functional with three Dunning basis sets: aug-cc-pVDZ, aug-cc-pVTZ, and aug-cc-pVQZ. Optimization of the complexes was done with the Boys–Bernardi full counterpoise method by Dannenberg [28,29]. Harmonic wavenumbers were calculated at both levels of theory.

## 3. Results and Discussion

In our previous study [17], a number of stable structures of the GA…N_2_ were identified by computational methods. However, only one complex form (SSC1) was experimentally observed after deposition in solid Ar matrix. The SSC1 complex involved a weak hydrogen bond between carboxylic OH group and nitrogen molecule. Interestingly, no complexes with N_2_ molecules attached to the alcoholic OH group could be identified.

### 3.1. Site-Selective Changes of SSC1

**Figure 2:**
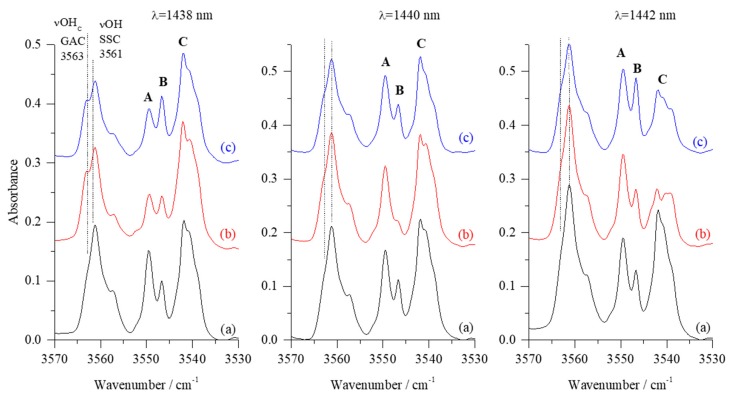
The νOH regions of the infrared spectra obtained for the 1:1 complex formed between SSC and N_2_ (SSC1): (**a**) After co-deposition GA with N_2_/Ar = 1/4000 at 18 K (10 K for measurement); (**b**) upon 360 min irradiation at 1438, 1440, or 1442 nm; (**c**) upon annealing at 33 K/10 K. The perpendicular broken lines show position of the bands due to the GA monomer species. A, B and C stand for different trapping sites of GA…N_2_.

For freshly deposited GA/N_2_/Ar matrix, the SSC1 complex exhibited a changing site structure for the carboxylic OH stretching mode depending on the deposition temperature used [17]. Figure 2 shows the νOH stretching regions of the SSC monomer and the SSC1 complex in the matrix obtained after co-deposition of GA with N_2_/Ar=1/4000 mixture at 18 K (measurement at 10 K) (traces (a), black). Thereafter, matrix samples were subjected to irradiation at the 1442 nm (6935 cm^−1^), 1440 nm (6944 cm^−1^), or 1438 nm (6954 cm^−1^), as shown as traces (b) in Figure 2. 

Different wavelengths were tested in order to induce isomerization in the SSC1 complex, but the most pronounced changes were observed for the above-mentioned wavelengths. The values correspond approximately to the first overtones of the stretching vibration of the alcoholic and carboxylic hydroxyl groups (2νOH_A_ and 2νOH_C_ of the SSC conformer) [6]. After irradiations, the matrices were annealed for 10 min at 33 K and next spectra were taken again at 10 K (traces (c), blue). The SSC1 complex was found to be isolated in different trapping sites, which manifested themselves by presence of the multiple bands in νOH, δOH, and other regions of the spectra [17]. We label these bands in the νOH region as A (3549.5 cm^−1^), B (3546.5 cm^−1^), and C (doublet at 3542.0/3540.0 cm^−1^).

Depending on the irradiation wavelength employed, various components of the νOH absorption originating from the SSC1 complex were differently affected. Irradiation at 1438 nm led to a strong decrease of intensity of the A band (*ca*. 60%), and the B band decreased less (*ca*. 30%). The intensity of the C doublet was almost unchanged, but a broadening at *ca.* 3539 cm^−1^ appeared. The irradiation at 1438 nm led also to decrease of the SSC monomer absorption and appearance of a shoulder at 3563.0 cm^−1^. The latter change could be expected at 1438 nm because of the proximity of the applied wavelength to that reported for the SSC→GAC isomerization of the monomeric species [4,6]. After irradiation, upon annealing at 33 K, intensity of the B component was recovered, whereas the A component remained unchanged. Simultaneously, both the νOH band of SSC monomer and the C doublet slightly decreased.

When irradiation was performed with 1440 nm instead of 1438 nm, the B component almost completely vanished, the A and C bands were very slightly affected, and SSC monomer absorption remained unchanged. Upon annealing at 33 K, the B band intensity was fully restored, whereas both the SSC monomer and the C doublet slightly decreased. In turn, irradiation at 1442 nm affected only the C doublet, which decreased in intensity by *ca.* 50% compared with the spectrum after deposition. This absorption was partially restored upon annealing, and a shoulder at 3539.0 cm^−1^ appearing in the spectra upon all three irradiations became better shaped. Additionally, the B band also gained intensity upon annealing.

The site-selective changes of the νOH components of the SSC1 absorption described above allow for some conclusions on trapping sites in which the SSC1 complex reside in the studied matrices. Comparing the dimensions of the SSC1 molecule (approximately 7.1 × 3.1 × 1.8 Å, estimated from computations) with the single substitutional site, i.e., molecule substituting one argon atom in the argon lattice (site diameter *ca.* 3.76 Å [30]), one may suppose that the complex can reside either in a relatively tight double substitutional site or in a loose site formed by substituting three or more argon atoms. The changes in intensity observed for the three components of the νOH absorption of SSC1 indicate that the applied energy through the 2νOH overtones is sufficient to induce dissociation of the weak hydrogen bond present in this complex. If SSC1 is in a tight site (represented by B component of the νOH absorption at 3546.5 cm^−1^), the changes observed could be due to the attached N_2_ molecule providing a more efficient energy dissipation channel, leading to elongation of the weak hydrogen bond. This can be understood as that the irradiation is able to detach the N_2_ molecule from the SSC subunit, and the annealing relaxes the local structure, again yielding the original complex. Therefore, the tight site does not result to rotamerisation that is expected upon NIR excitation. On the contrary, when the complex is in a loose site (represented by A or C components at 3549.5 cm^−1^ and 3542.0/3540.0 cm^−1^), the rotamerisation is more viable to take place, and the nitrogen molecule in close vicinity forms molecular complex with the new GA rotamer.

Similar wavelength-dependent changes of the SSC1 species were detected also in the in-plane-deformation δOH region of the spectra (see Figure S1 in Supporting Information).

### 3.2. Photoproducts Formed Upon Vibrational Overtone Excitation

**Figure 3:**
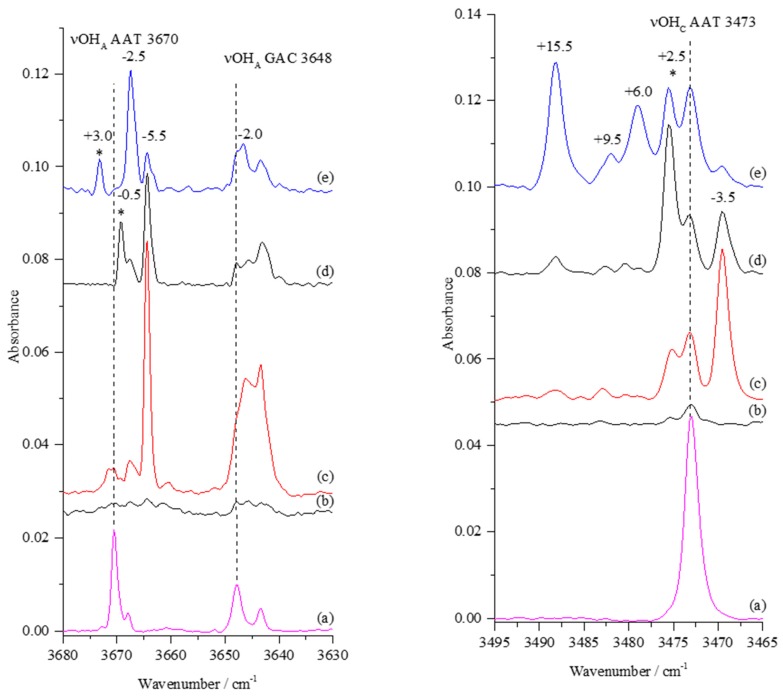
The νOH regions of the infrared spectra of GAC and AAT monomers and their complexes with nitrogen: (**a**) GA/Ar after deposition at 18 K and two subsequent irradiations at 1438 and 1400 nm; (**b**) after co-deposition, GA with N_2_/Ar=1/4000 at 18 K (10 K for measurement); (**c**–**e**) upon 360 min irradiation at 1438, 1440, or 1442 nm, respectively. The perpendicular broken lines show position of the GA monomer bands. Stars (*) denote bands disappearing upon annealing.

Due to a very low abundance of two higher energy conformers of GA (GAC and AAT), the νOH bands arising from these monomers and their complexes with nitrogen, expected in the 3670–3640 and 3490–3460 cm^−1^ regions, were negligible in the spectra of matrices obtained directly after deposition. Selected regions of the GA/N_2_/Ar spectra after deposition are presented in Figure 3 and Figure S2 (traces (b)) and compared with irradiated GA/Ar matrix (trace (a)).

The positions of bands expected for the AAT and GAC monomers are indicated by perpendicular dashed lines. Upon irradiation of the GA/N_2_/Ar matrices, new spectral features grew, as shown in the resultant spectra (traces c, d, and e in Figure 3, respectively). Based on the changes in the νOH regions, the amount of the GAC and AAT monomers formed upon irradiation was small. This implies that the relatively strong band at 3473 cm^−1^ present in the spectra of the irradiated matrices is not due to the νOH_C_ of the AAT monomer only, but encompass a newly formed complex with N_2_ as well. Therefore, all new bands detected in the νOH_A_ and νOH_C_ regions could be assigned to the GAC and AAT complexes with nitrogen molecule. Table 1 shows the positions of the bands appearing in the νOH regions of the GA/N_2_/Ar spectra upon irradiations and annealing, together with their shifts relative to the corresponding monomer absorptions.

Upon 1438 nm irradiation, simultaneously with the decrease of the bands A and B of the SSC1 complex (Figure 2), new bands at 3664.5, ca. 3646 (a broad doublet), 3475.5, 3473.0, and 3469.5 cm^−1^ appeared in the spectra. Other changes were observed when 1440 nm irradiation was used that induced a slight decrease of A and C bands with vanishing of the B component in the νOH_C_ region of SSC1. Simultaneously, the bands at 3664.5, 3646, and 3469.5 cm^−1^ were much weaker than those detected upon 1438 nm irradiation but absorptions at 3669.5, 3488.5, and 3475.5 cm^−1^ either appeared or increased in their intensity. In turn, upon 1442 nm irradiation (where mostly the C component of SSC1 was affected), new bands appeared or increased at 3673.0, 3667.5, 3488.5, 3479.0 cm^−1^, whereas those present earlier at 3664.5 and 3469.5 cm^−1^ were hardly seen.

**Figure 4:**
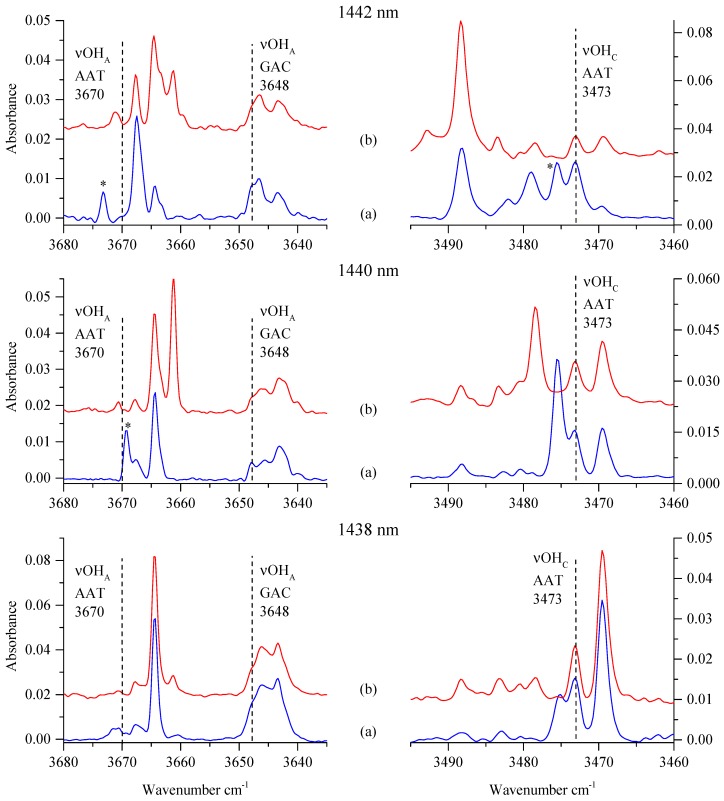
The νOH regions of the infrared spectra of GAC and AAT complexes with nitrogen: (**a**) GA/N_2_/Ar deposited at 18 K/10 K and after 360 min irradiation at 1438, 1440, or 1442 nm; (**b**) the same matrix upon annealing at 33 K/10 K. The perpendicular broken lines show position of the GA monomer bands. Stars (*) denote bands disappearing upon annealing.

The overall picture observed in the described νOH_A_ and νOH_C_ regions of newly formed complexes is different depending on the wavelength used for irradiation, and therefore they are connected with the local site structure (described by the bands A, B, or C, Figure 2), where the precursor SSC1 was situated. Figure 4 shows the behavior of the νOH vibrational bands of the newly formed complexes after irradiation and upon annealing (33 K, 10 min).

Annealing of the GA/N_2_/Ar matrices after irradiation with 1438 nm did not much influence the infrared spectra. This suggests that new complexes characterised by absorptions at 3664.5 and *ca*. 3646 and 3469.5 cm^−1^ were formed in stable matrix sites. Different outcomes were observed after annealing of the sample irradiated with 1440 and 1442 nm. In these cases, positions and intensities of bands appearing due to new complexes showed clear changes upon annealing. The 3669.5 and 3475.5 cm^−1^ bands present after the 1440 nm irradiation decreased in intensity in favor of a new one appearing at 3661.0 and 3478.5 cm^−1^, respectively. Based on the annealing behavior of the 3669.5 and 3475.5 cm^−1^ bands, one can assign them to the GA…N_2_ complex situated in an unstable matrix site. The new bands at 3673.0 and 3479.0 cm^−1^ generated upon 1442 nm irradiation decreased or bleached out upon annealing. This indicates the presence of an additional unstable site. Similar conclusions were drawn for bands observed during the Raman spectroscopy study [16] of the GA/N_2_/Ar matrices (see Table 1). 

The most intense vibrational band after 1442 nm irradiation was found in the νOH_C_ region at 3488.5 cm^−1^ with a weak counterpart in the νOH_A_ region at 3663.5 cm^−1^. These features behaved differently than those described above, since their intensity increased upon annealing, and as such, they are considered to be connected with a GA…N_2_ complex situated in a stable matrix site.

### 3.3. Structures of the Newly Formed Complexes

As shown in Figure 3 and Figure 4, the spectral picture of the irradiated GA/N_2_/Ar matrices depends on the wavelength used for irradiation, as well as on the annealing of the matrices. Table 1 presents the positions of the described sets of bands appearing or changing upon irradiations at various wavelengths or annealing. They differ by several wavenumbers, showing that they may belong either to different structures of the AAT…N_2_ and GAC…N_2_ complexes or to the same species formed in different matrix sites.

The assignment of the newly formed bands presented in Table 1 is supported by the results of the calculations performed at different levels of theory. The calculated shifts of the νOH and νC=O modes and their intensities obtained for nine GA complexes with nitrogen are shown in Table 2 and Table S1. Analysis of these shifts indicate that for such weak complexes, more demanding computations are probably needed in order to obtain more satisfactory agreement with the experimental data However, among the methods used here, the B3LYPD3 calculations with relatively good basis sets give quite consistent results compared with experimental spectra [31].

The first question to answer is whether both AAT and GAC complexes with N_2_ are formed in the irradiated/annealed matrices. This question is particularly relevant in the context of the recently published results of the GA…N_2_ complexes studied by Raman spectroscopy [16], which indicate that only the AAT…N_2_ complex is present in argon matrices. The analysis of the νC=O region of the GA/N_2_/Ar matrices obtained upon irradiation at 1438, 1440, and 1442 nm (Figure S2 and Table S2 in Supporting Information) reveals that both AAT (1806 cm^−1^) and GAC (1784 cm^−1^) carbonyl stretching vibrations were disturbed by the presence of nitrogen, as shown by broadening/shift of the corresponding monomer νC=O bands. Thus, it is plausible that both AAT and GAC conformers are present and involved in formation of complexes with N_2_.

The three bands in the alcoholic stretching region (νOH_A_) at 3673.0, 3669.5, and 3667.5 cm^−1^, that appeared upon irradiation both at 1440 and 1442 nm wavelengths, have a common feature. They either vanished or decreased in intensity upon annealing of the matrices at 33 K and contributed to the growth of a new band at 3661.0 cm^−1^. Similar behavior was found for the carboxylic OH stretching vibration (νOH_C_). The two bands at 3479.0 and 3475.5 cm^−1^ decreased/vanished in favor of the absorptions at 3483.5 and 3478.5 cm^−1^. Relative to the AAT monomer, the vibrational shifts observed for the bands found after annealing were –9 cm^−1^ and +10.5 or +5.5 cm^−1^, respectively, for νOH_A_ and νOH_C_ modes (see Table 1). According to computations, the shifts predicted for the AAT1 structure fit relatively well to these experimental findings. Similarly, the absorptions at 3663.5 (νOH_A_) and 3488.5 cm^−1^ (νOH_C_) with the shifts of –6.5 and +15.5 cm^−1^ are assigned to the AAT1 formed in a stable matrix site (see chapter 3.2).

The two vibrational absorptions at 3664.5 cm^−1^ (νOH_A_) and 3469.5 cm^−1^ (νOH_C_) were most profound after irradiation using 1438 nm wavelength, and annealing led to a slight increase of intensity of these bands without changes of their position. The experimental wavenumber shifts relative to the AAT monomer are equal to –5.5 and –3.5 cm^−1^ for the νOH_A_ and νOH_C_ stretching vibrations, respectively. These values are in reasonable agreement with those predicted theoretically for the AAT3 complex. Looking at the νOH_C_ region of the SSC1 complex (Figure 2), one can notice that upon 1438 nm irradiation, the band A at 3549.5 cm^−1^ was irreversibly bleached, suggesting that the AAT3 complex was formed from the SSC1 precursor occupying the matrix cage characterised by the A band.

Figure S3 presents kinetic profiles obtained for selected bands arising from the newly formed GA complexes with nitrogen (AAT1 and AAT3). It shows an evolution of concentration of the species during irradiation and upon annealing. The changes upon annealing clearly demonstrate the local site structure differences between AAT1 and AAT3 based on their different response. A molecular complex in a looser site is more easily interconverted upon annealing. 

Based on the observed changes in the νC=O stretching region (See Figure S2), we also considered the presence of GAC complexes in the matrices upon irradiation. A broad absorption between 3640–3650 cm^−1^ observed close to the νOH_A_ of the GAC monomer can be assigned as the GAC1 complex. The shifts of the OH stretching vibrations predicted for this structure at B3LYPD3 level with different basis sets are about –1 cm^−1^ and in the range from –35 to –39 cm^−1^ for the νOH_A_ and νOH_C_ modes, respectively. Comparing these with the corresponding experimental values of –2 and –24 cm^−1^ suggests tentative assignment of these bands to the GAC1 structure.

## 4. Conclusions

**Figure 5:**
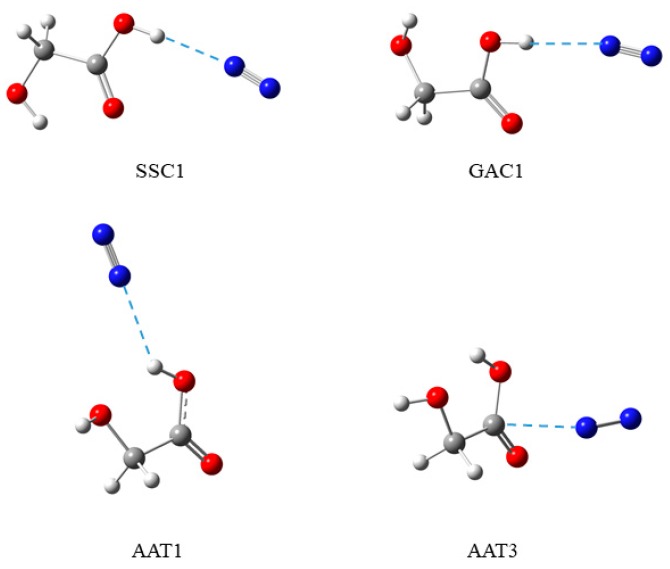
Computational structures of four glycolic acid complexes with nitrogen identified in argon matrices.

Phototransformations of the SSC1 complex formed between the most stable form of glycolic acid (SSC) and nitrogen molecule, appearing upon the νOH overtone excitation at 1438, 1440, and 1442 nm, turned out to be quite complicated processes. First, we observed site-selective response of the SSC1 species isolated in various trapping sites of an argon matrix when different wavelengths for the IR-irradiation were used. The changes were followed in the stretching vibration region of the OH group of the carboxylic group νOH_C_. Simultaneously, new complexes were detected upon irradiations formed between two less stable conformers of glycolic acid (AAT and GAC) and nitrogen. Comparison of the theoretically predicted shifts of the νOH_A_, νOH_C_, and νC=O modes with the experimental results allowed identification of two conformers of the AAT complex with nitrogen (AAT1 and AAT3), and more tentatively one structure formed by GAC (GAC1). These structures are pictured in Figure 5 based on theoretical calculations.

Our experimental and computational results show that glycolic acid complexes with nitrogen are extremely sensitive on changes in the matrix surroundings. The most efficient formation of the AAT3 and GAC1 complexes took place upon 1438 nm irradiation, whereas the AAT1 population was the highest in the matrices subjected to 1442 nm radiation. This indicates that even a small change in the irradiation wavelength can have important consequences on the isomerisation processes, as well as the local site structure of the formed photoproducts. 

## Figures and Tables

**Table 1:** Experimental positions of the νOH bands and their shifts (in cm^−1^) observed for GA…N_2_ complexes in argon matrices.

Infrared		Assignment	Raman [16]	
νOH_A_^a^	ΔνOH_A_		νOH_A_	ΔνOH_A_
3673.0↓/3661.0	+3.0/–9.0	AAT1	3672↓	–3
3669.5↓/3661.0	–0.5/–9.0	AAT1	3669	–6
3667.5↓/3661.0	–2.5/–9.0	AAT1		
3664.5↑	–5.5	AAT3		
3663.5↑ sh	–6.5	AAT1		
*ca.* 3646 ^b^	–2	GAC1		
			3567↓	
3557.0	–4.0	SSC1	3562	–4
νOH_C_	ΔνOH_C_		νOH_C_	ΔνOH_C_
3549.5	–11.5	SSC1	3554	–12
3546.5	–14.5	SSC1		
3542.0/3540.0	–19.0/–21.0	SSC1	3545	–21
3539.0 ^c^	–24.0	GAC1		
3488.5↑	+15.5	AAT1	3487↓/3486	+9/+8
3479.0↓/3483.5	+6.0/+10.5	AAT1	3479↓	+1
3475.5↓/3478.5	+2.5/+5.5	AAT1	3473	–5
3473.0	0			
3469.5↑	–3.5	AAT3		

^a^ Up and down arrows indicate intensity changes upon annealing; band positions before/after annealing. ^b^ Broad absorption. ^c^ Observed as a shoulder on the νOH_C_ absorption of SSC1 (see Figure 2). Experimental IR positions of SSC νOH_A_ and νOH_C_ is 3561.0; GAC: νOH_A_ 3648.0; νOH_C_ 3563.0; AAT: νOH_A_ 3670.0; νOH_C_ 3473.0 cm^−1^. Experimental Raman spectra positions of SSC νOH_A_ and νOH_C_ is 3566.0; GAC: νOH_A_ 3652.0, νOH_C_ 3568.0; AAT: νOH_A_ 3675.0, νOH_C_ 3478.0 cm^−1^ [12].

**Table 2:** The νOH shifts (in cm^−1^) and intensities (in km mol^−1^, given in parentheses) in GA complexes with nitrogen at different levels of theory.

		**SSC1**		**SSC2**		**SSC3**	
		νOH_A_	νOH_C_	νOH_A_	νOH_C_	νOH_A_	νOH_C_
**MP2**	6-311++G(2d,2p)	−5(79)	−30(302)	3(119)	1(117)	0(32)	0(140)
**B3LYPD3**	6-311++G(2d,2p)	−4(74)	−37(309)	8(138)	0(84)	−1(77)	0(73)
	aug-cc-pVDZ	−5(69)	−37(303)	9(143)	0(70)	−1(79)	0(73)
	aug-cc-pVTZ	−4(72)	−40(316)	9(142)	1(79)	−1(73)	0(70)
	aug-cc-pVQZ	−4(73)	−40(314)	9(145)	0(78)	0(71)	0(73)
		**GAC1**		**GAC2**		**GAC3**	
		νOH_A_	νOH_C_	νOH_A_	νOH_C_	νOH_A_	νOH_C_
**MP2**	6-311++G(2d,2p)	−2(58)	−31(293)	−6(158)	−3(88)	1(56)	−2(93)
**B3LYPD3**	6-311++G(2d,2p)	−1(49)	−35(298)	−4(155)	−2(72)	1(48)	0(78)
	aug-cc-pVDZ	−1(45)	−35(288)	−4(152)	−2(69)	1(45)	0(74)
	aug-cc-pVTZ	−1(47)	−39(307)	−6(164)	−2(69)	1(47)	0(75)
	aug-cc-pVQZ	−1(48)	−39(305)	−6(165)	−3(69)	1(47)	0(76)
		**AAT1**		**AAT2**		**AAT3**	
		νOH_A_	νOH_C_	νOH_A_	νOH_C_	νOH_A_	νOH_C_
**MP2**	6-311++G(2d,2p)	−1(67)	−1(233)	−34(88)	−20(152)	0(72)	−4(170)
**B3LYPD3**	6-311++G(2d,2p)	−4(57)	10(211)	−22(81)	1(128)	−3(63)	−1(139)
	aug-cc-pVDZ	−4(52)	12(201)	−19(76)	1(120)	−2(58)	−1(131)
	aug-cc-pVTZ	−5(54)	11(211)	−22(79)	1(122)	−3(60)	−1(133)
	aug-cc-pVQZ	−4(55)	11(212)	−22(79)	1(122)	−3(61)	0(133)

## Supplementary Materials

The following are available online: Figure S1. The δOH regions of the infrared spectra obtained for the 1:1 complex formed between SSC and N_2_ (SSC1): (**a**) After co-deposition GA with N_2_/Ar=1/4000 at 18 K/10 K; (**b**) upon 360 min irradiation at 1438, 1440, or 1442 nm; (**c**) upon annealing at 33 K/10 K; Figure S2. The νC=O region of the infrared spectra of SSC, GAC, and AAT monomers and their complexes with nitrogen: (**a**) After deposition GA/Ar at 18 K/10 K and two subsequent irradiations at 1438 and 1400 nm; (**b**) after co-deposition GA with N_2_/Ar=1/4000 at 18 K/10 K; (c,d,e) upon 360 min irradiation at 1438, 1440, or 1442 nm, respectively; Figure S3. Plots of the integrated intensity of the selected bands of AAT1 and AAT3 versus time of irradiation; Table S1. The νC=O shifts and intensities in GA complexes with nitrogen at different levels of theory; Table S2. Experimental positions of the νC=O bands and their shifts observed for GA…N_2_ complexes in argon matrices.
