# Supplementary material for: Complexes of Glycolic Acid with Nitrogen Isolated in Argon Matrices. II. Vibrational Overtone Excitations
Source: Molecules. 2019 Sep 6;24(18):3245. doi: 10.3390/molecules24183245 (PMC6766840; doi:10.3390/molecules24183245)
Supplement: Supplementary file 1 [file molecules-24-03245-s001.pdf]

Supporting Information

Complexes of glycolic acid with nitrogen isolated in argon matrices. II. Vibrational overtone excitations

Iwona Kosendiak, Jussi Ahokas, Justyna Krupa, Jan Lundell, Maria Wierzejewska

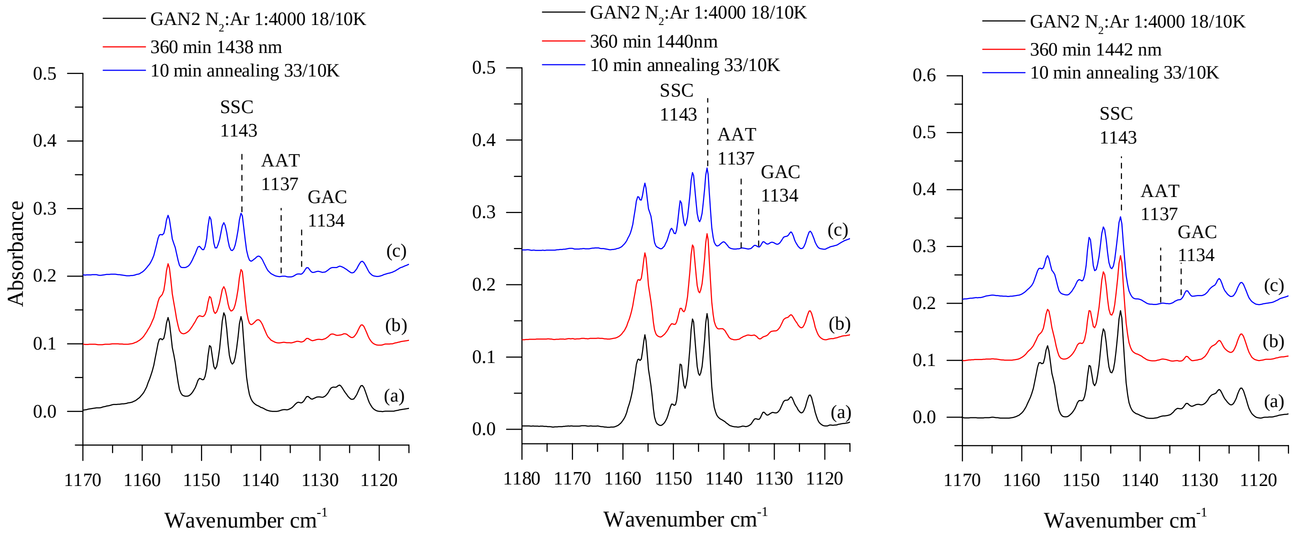

Figure S1. The δOH regions of the infrared spectra obtained for the 1:1 complex formed between SSC and N_2 (SSC1): (a) after co-deposition GA with N_2/Ar=1/4000 at 18 K (10 K for measurement); (b) upon 360 min irradiation at 1438, 1440 or 1442 nm; (c) upon annealing at 33 K/10 K. The perpendicular broken lines show position of the bands due to the GA monomer species.

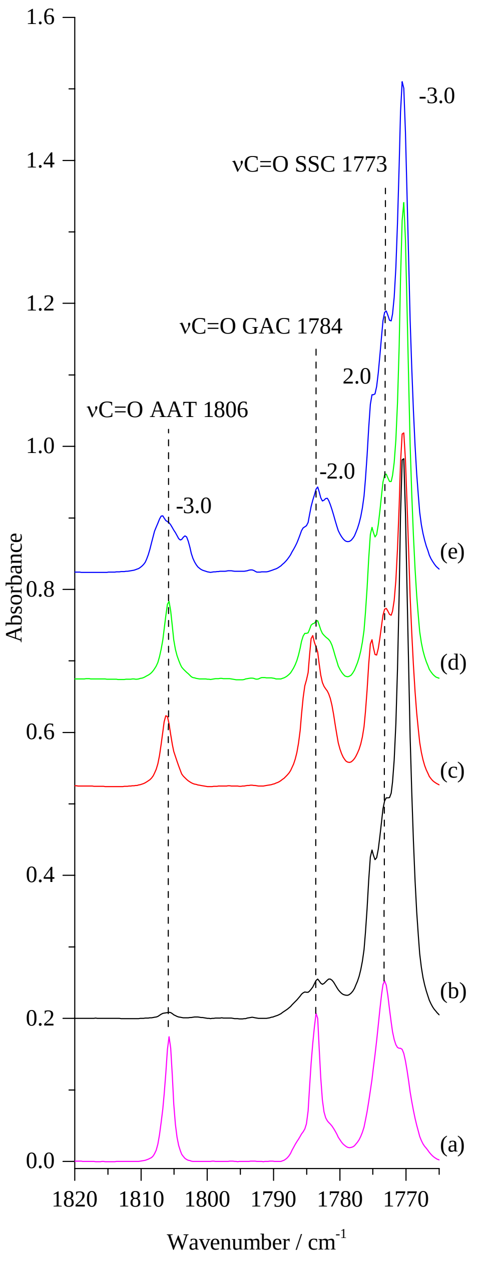

Figure S2. The νC=O region of the infrared spectra of SSC, GAC, AAT monomers and their complexes with nitrogen: (a) after deposition GA/Ar at 18 K/10 K and two subsequent irradiations at 1438 and 1400 nm (b) after co-deposition GA with N_2/Ar=1/4000 at 18 K/10 K; (c,d,e) upon 360 min irradiation at 1438, 1440 or 1442 nm, respectively. The perpendicular broken lines show position of the GA monomer species.

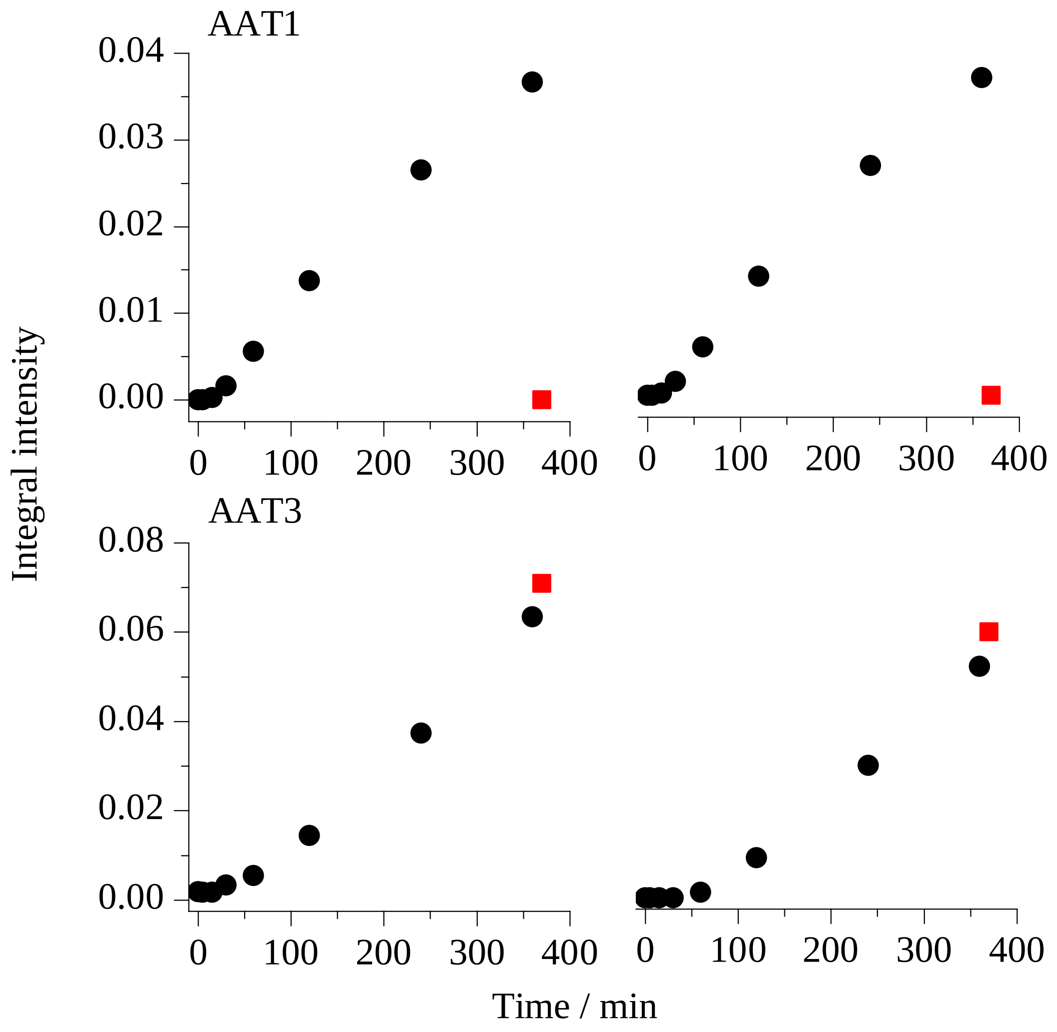

Figure S3. Plots of the integrated intensity of the selected bands of AAT1 (νOH$_A$ 3669.5 and νOH$_C$ 3475.5 cm^{-1}, λ=1440 nm) and AAT3 (νOH$_A$ 3664.5 and νOH$_C$ 3469.5 cm^{-1}, λ=1438 nm) versus time of irradiation. The last points (red squares) relate to the annealing at 33 K (10 K for measurement).

Table S1. The ◎C=O shifts and intensities in GA complexes with nitrogen at different levels of theory.

		SSC1	SSC2	SSC3
		νC=O	νC=O	νC=O
MP2	6-311++G(2d,2p)	-5(246)	1(274)	-1(249)
B3LYPD3	6-311++G(2d,2p)	-6(297)	1(338)	0(301)
	aug-cc-pVDZ	-7(284)	0(326)	-2(289)
	aug-cc-pVTZ	-6(289)	1(332)	0(294)
	aug-cc-pVQZ	-8(290)	0(333)	-2(295)
		GAC1	GAC2	GAC3
		νC=O	νC=O	νC=O
MP2	6-311++G(2d,2p)	-4(243)	-4(267)	1(245)
B3LYPD3	6-311++G(2d,2p)	-5(297)	-4(325)	1(301)
	aug-cc-pVDZ	-5(281)	-4(310)	1(285)
	aug-cc-pVTZ	-6(289)	-4(320)	2(293)
	aug-cc-pVQZ	-6(290)	-4(320)	2(294)
		AAT1	AAT2	AAT3
		νC=O	νC=O	νC=O
MP2	6-311++G(2d,2p)	-4(276)	-2(247)	0(258)
B3LYPD3	6-311++G(2d,2p)	-5(320)	-2(295)	-1(295)
	aug-cc-pVDZ	-5(306)	-1(281)	-1(281)
	aug-cc-pVTZ	-5(319)	-1(292)	-1(292)
	aug-cc-pVQZ	-5(319)	-1(292)	-1(293)

Table S2. Experimental positions of the ◎C=O bands and their shifts (cm^{-1}) observed for GA⋯N_2 complexes in argon matrices.

Infrared		Assignment	Raman [16]	
νC=O	ΔνC=O		νC=O	ΔνC=O
1805.5	-0.5	AAT3?		
1803.0	-3.0	AAT1	1810	0
1782.0	-2.0	GAC1	1787	-1
1775.0	2.0	SSC1?		
1770.0	-3.0	SSC1	1775	-2

Experimental IR positions of SSC νC=O 1773.0 cm^{-1}, GAC: νC=O 1784.0 cm^{-1}, AAT: νC=O 1806.0 cm^{-1}.
Experimental Raman positions of SSC νC=O 1777.0 cm^{-1}, GAC: νC=O 1788.0 cm^{-1}, AAT: νC=O 1810.0 cm^{-1} [12]
